# The oxidative stress response of *Streptococcus pneumoniae*: its contribution to both extracellular and intracellular survival

**DOI:** 10.3389/fmicb.2023.1269843

**Published:** 2023-09-13

**Authors:** Mirelys Hernandez-Morfa, Nadia B. Olivero, Victoria E. Zappia, German E. Piñas, Nicolas M. Reinoso-Vizcaino, Melina B. Cian, Mariana Nuñez-Fernandez, Paulo R. Cortes, Jose Echenique

**Affiliations:** ^1^Centro de Investigaciones en Bioquímica Clínica e Inmunología (CIBICI-CONICET), Facultad de Ciencias Químicas, Universidad Nacional de Córdoba, Córdoba, Argentina; ^2^Departamento de Bioquímica Clínica, Facultad de Ciencias Químicas, Universidad Nacional de Córdoba, Córdoba, Argentina; ^3^Centro de Química Aplicada, Facultad de Ciencias Químicas, Universidad Nacional de Córdoba, Córdoba, Argentina

**Keywords:** *Streptococcus pneumoniae*, oxidative stress, two-component systems, intracellular survival, immune cells, influenza A, persistence, fluoroquinolones

## Abstract

*Streptococcus pneumoniae* is a gram-positive, aerotolerant bacterium that naturally colonizes the human nasopharynx, but also causes invasive infections and is a major cause of morbidity and mortality worldwide. This pathogen produces high levels of H_2_O_2_ to eliminate other microorganisms that belong to the microbiota of the respiratory tract. However, it also induces an oxidative stress response to survive under this stressful condition. Furthermore, this self-defense mechanism is advantageous in tolerating oxidative stress imposed by the host’s immune response. This review provides a comprehensive overview of the strategies employed by the pneumococcus to survive oxidative stress. These strategies encompass the utilization of H_2_O_2_ scavengers and thioredoxins, the adaptive response to antimicrobial host oxidants, the regulation of manganese and iron homeostasis, and the intricate regulatory networks that control the stress response. Here, we have also summarized less explored aspects such as the involvement of reparation systems and polyamine metabolism. A particular emphasis is put on the role of the oxidative stress response during the transient intracellular life of *Streptococcus pneumoniae*, including coinfection with influenza A and the induction of antibiotic persistence in host cells.

## Introduction

*Streptococcus pneumoniae* (or the pneumococcus) is an aerotolerant pathogen that colonizes the human nasopharynx. In fact, it is part of the commensal microbiota found in the human upper respiratory tract (Bogaert et al., 2004), where this pathogen can be isolated from 5–90% of healthy individuals, depending on the range age of the population (Gierke et al., 2021). This asymptomatic transitory phase can lead to various pneumococcal diseases when bacteria migrate to the lower respiratory tract (Subramanian et al., 2019). *Streptococcus pneumoniae* can cause both minor infections, such as otitis and sinusitis, and severe invasive infections, such as community-acquired pneumonia and meningitis. It has been reported that around 10% of patients with invasive pneumococcal disease die from their illness (CDC, 2012). Regardless of the availability of antibiotics and vaccines, pneumococcal diseases remain the leading cause of death among vaccine-preventable diseases worldwide. According to the World Health Organization (WHO), these diseases cause over 1 million deaths annually.^1^

*Streptococcus pneumoniae* employs various adaptive mechanisms to survive in the mucosa of the respiratory tract and cause disease. It can tolerate different levels of oxygen in aerobic conditions. In the nasopharynx and lungs, the pneumococcus is exposed to a partial pressure of oxygen that is similar to that of the atmosphere and thrives in micro-aerophilic conditions in the lower respiratory tract or middle ear. However, during more severe infections like bacteremia and meningitis, it must also be able to survive in anaerobic conditions.

In addition to naturally stressful environments, the pneumococcus is also subjected to oxidative conditions imposed by the host’s immune response. In response to a bacterial infection, the body’s first line of defense involves the migration of leukocytes and the generation of chemokines and cytokines. In this context, polymorphonuclear leukocytes produce hydrogen peroxide (H_2_O_2_), superoxide anion radicals (O_2_−∙), and nitric oxide (NO∙). Importantly, superoxide anion radicals and nitric oxide can combine to form peroxynitrite (ONOO), a potent oxidant with cytotoxic effects (Barichello et al., 2013).

In this review, we will summarize how the exceptional endogenous production of H_2_O_2_ affects the oxidative stress response (OSR) of this pathogen. We will also review the molecular defense mechanisms that *S. pneumoniae* has developed to protect itself against oxidative stress induced by human tissues. Furthermore, we will outline the impact of metal transport and signal transduction systems on the development of oxidative stress response. Finally, we focus on the relevance of the pneumococcal OSR during the transient intracellular life in host cells, as well as the emergence of an antibiotic persistence induced by oxidative conditions, and the risk to generate antibiotic resistance, a scenario that complicates the antimicrobial therapy of pneumococcal diseases.

## Generation of endogenous H_2_O_2_

*Streptococcus pneumoniae* exhibits the ability to tolerate and metabolize oxygen, producing high levels of H_2_O_2_ as a byproduct that can accumulate to millimolar concentrations, providing it with a competitive edge over other commensal and pathogenic bacteria in the upper respiratory tract (Pericone et al., 2000). Moreover, the H_2_O_2_ exposure induces toxic DNA double-strand breaks that precede apoptosis in alveolar cells. These genotoxic and cytotoxic effects are directly implicated in the pathogenesis of host cellular injury in pneumococcal pneumonia (Duane et al., 1993; Rai et al., 2015).

Endogenously generated H_2_O_2_ by the pneumococcus can rapidly diffuse through cell membranes, accumulate in the extracellular milieu, and eliminate other microorganisms, thereby promoting colonization and worsening virulence (Pericone et al., 2000). The hyperproduction of H_2_O_2_ is approximately 10^3^ times higher than the level that inhibits the growth of *Escherichia coli* cells that lack the ability to scavenge H_2_O_2_ (Seaver and Imlay, 2001). Concentrations between 0.1 and 1.0 mM H_2_O_2_ are capable to kill or inhibit *in vitro* other respiratory tract pathogens, such as *Moraxella catarrhalis*, *Haemophilus influenzae*, and *Neisseria meningitidis*. Despite producing catalase, an important metabolic enzyme used to eliminate H_2_O_2_, these bacterial species were found to be sensitive to H_2_O_2_ concentrations produced by *S. pneumoniae* (Pericone et al., 2000).

**Figure 1:**
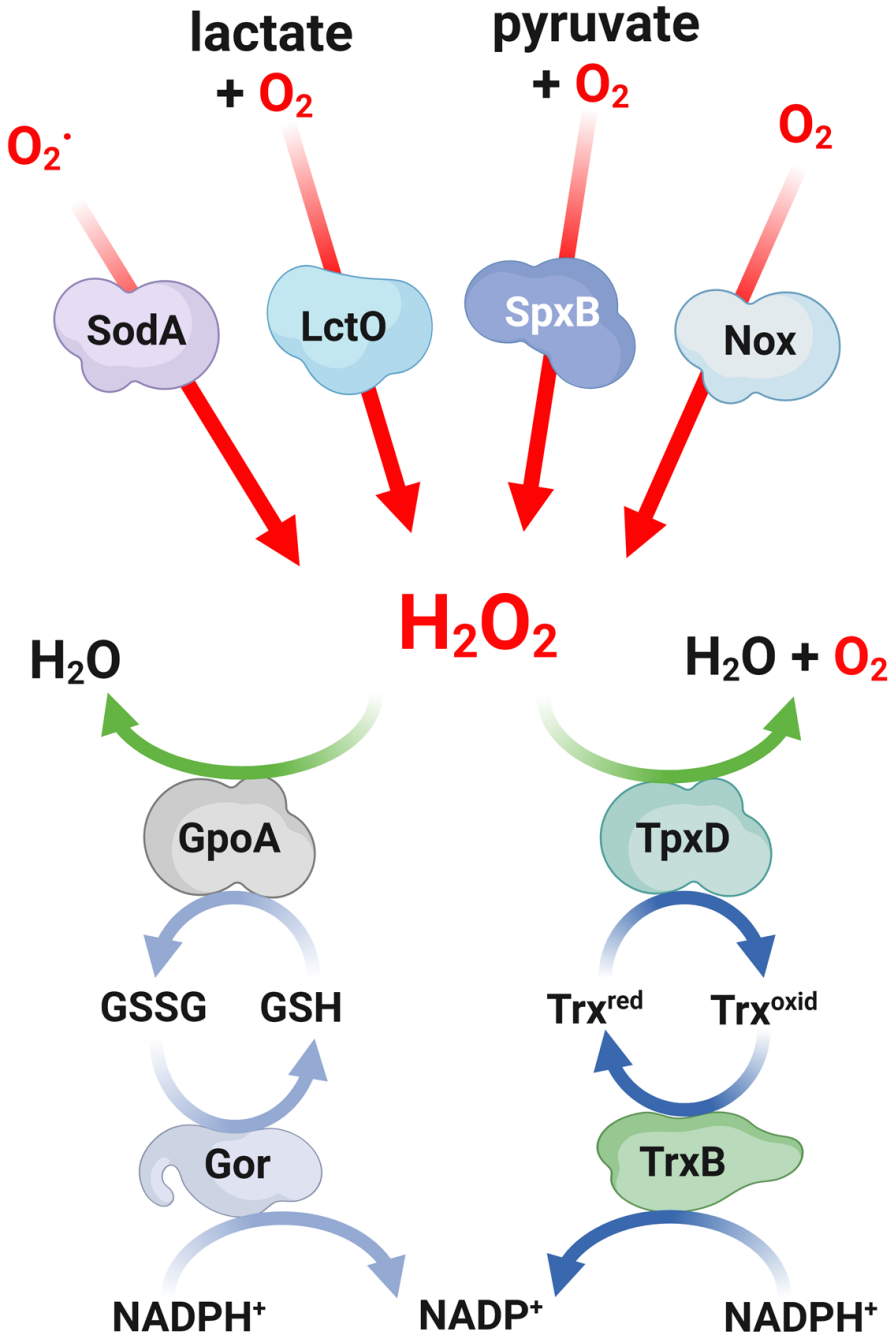
Mechanisms involved in both production and defense against H_2_O_2_ in *Streptococcus pneumoniae*. Scheme of the mechanisms involved in both the production and defense against H_2_O_2_ in *S. pneumoniae*. Red lines indicate the enzymatic reactions involved in the production of H_2_O_2_, while green lines represent the pathways for detoxifying H_2_O_2_. The light blue and blue lines show the redox reactions that involve glutathione and peroxiredoxins, respectively. H_2_O_2_, hydrogen peroxide; O_2_, superoxides; SodA, superoxide dismutase; LctO, lactate oxidase; SpxB, pyruvate oxidase; Nox, NADH oxidase; GpoA, glutathione peroxidase; Gor, glutathione reductase; TpxD, thiol peroxidase; TrxB, thioredoxin reductase.

One of the metabolic pathways utilized by pneumococci to process O_2_ involves the production of H_2_O_2_. The SpxB pyruvate oxidase is responsible for over 80% of endogenous H_2_O_2_ synthesis, which is generated by converting pyruvate to acetyl phosphate and releasing H_2_O_2_ (Pericone et al., 2000; Lisher et al., 2017). Because the SpxB reaction generates ATP, it was reported that the susceptibility of the *spxB* mutant to external 20 mM H_2_O_2_ was due to a rapid depletion of ATP that affected pneumococcal viability (Pericone et al., 2003). Furthermore, lactate oxidase (LctO) complements this oxidative reaction through the generation of pyruvate from lactate, consuming O_2_ and releasing H_2_O_2_. The biosynthesis of pyruvate by LctO facilitates the production of H_2_O_2_ through the SpxB pathway (Lisher et al., 2017; Figure 1).

The induction of apoptosis-like death during the stationary phase of *S. pneumoniae* is attributed to the presence of H_2_O_2_, which is mainly generated by SpxB, and confers a selective advantage in nasopharyngeal colonization. It seems that the *spxB* mutant strain is unable to thrive in this environment, unlike the wild-type strain (Regev-Yochay et al., 2007). In this sense, the *spxB* mutant exhibited impaired virulence in animal models for sepsis, pneumonia, and nasopharyngeal colonization, indicating that SpxB is a crucial factor in determining the virulence of *S. pneumoniae* (Spellerberg et al., 1996).

It has been suggested that the activity of pyruvate oxidase plays a crucial role in the response of *S. pneumoniae* to fluctuations in oxygen levels, regardless of exposure to external H_2_O_2_ (Carvalho et al., 2013). This specific activity is believed to regulate capsule production, which is controlled by the bacterium to adapt to its environment. The absence of SpxB results in various metabolic changes, including an upregulation of the *cps* operon, leading to excessive production of the capsule. These findings were confirmed in a mutant strain lacking *spxB* that exhibited lower levels of acetyl-CoA, an intermediary metabolite involved in capsule biosynthesis. This effect was observed only in certain serotypes that produce acetylated sugars. It was proposed that the reduced availability of acetyl-CoA is responsible for the decreased capsule production (Echlin et al., 2016).

It has been reported that *S. pneumoniae* has the capability to modulate the flow of pyruvate metabolism based on the specific type of carbohydrate present in its environment. In the presence of galactose, there is a shift in pyruvate metabolism toward the production of acetyl-CoA. Notably, the majority of acetyl-CoA produced by the enzymes SpxB and the pyruvate dehydrogenase complex is primarily utilized for the biosynthesis of the bacterial capsule. Conversely, the acetyl-CoA produced by pyruvate formate lyase serves the purpose of regenerating NAD+ (Echlin et al., 2020). These variations in capsule production have been found to have significant implications in assays *in vivo*, as they play a critical role in the pathogenesis of pneumococcal infections (Kim and Weiser, 1998).

## Detoxifying mechanisms of reactive oxygen species

Reactive oxygen species (ROS) are chemical entities that arise from oxygen metabolism, including peroxides and superoxides, which have a role in different cellular processes, such as cell signaling and homeostasis, and produce cellular damage. The generation of H_2_O_2_ is a crucial virulence determinant for *S. pneumoniae*. The production and discharge of high levels of H_2_O_2_ are capable of eliminating other bacterial pathogens and causing damage to human tissues during infection. Nevertheless, apart from the endogenous H_2_O_2_ production that serves as a metabolic weapon, the pneumococcal cells necessitate a potent stress response to endure oxidative conditions. Several oxidative gene mutants that have been examined thus far have demonstrated susceptibility to exogenous H_2_O_2_, implying their involvement in the OSR mechanism of *S. pneumoniae*. The first enzyme involved in H_2_O_2_ tolerance was SpxB, which is responsible for endogenous H_2_O_2_ generation. Clearly, SpxB has no a role as a detoxification mechanism, however, it is probable that SpxB-mediated H_2_O_2_ production facilitates the induction of OSR in *S. pneumoniae.*

The oxygen metabolism generates ROS, and these harmful compounds require the presence of catalases and peroxidases, as well as superoxide dismutases and reductases, for the detoxification of bacterial ROS (Mishra and Imlay, 2012). NADPH peroxidase and catalases are the most common H_2_O_2_ detoxifying enzymes in bacteria. However, the corresponding encoding genes have not been found in the genomes of streptococci, enterococci, and leuconostoc (Mishra and Imlay, 2012). Instead, *S. pneumoniae* expresses many other H_2_O_2_ scavengers, as reported in other bacterial genera, although the underlying reason for this enzymatic diversity remains unknown.

As described for peroxides, the control of superoxide levels is also relevant for bacterial survival and it is considered one of the major defense mechanisms against oxidative stress (McCord and Fridovich, 1968). In prokaryotes, superoxides are converted to peroxides by the action of superoxide dismutases. Metals can be used as cofactors for various enzymes that react with superoxides to produce H_2_O_2_, a less reactive compound that should be metabolized by other enzymes. To date, six types of bacterial superoxide dismutases have been characterized. This classification depends on the metal cofactor, such as SodA (MnSOD), SodB (FeSOD), SodC (CuSOD) (McCord and Fridovich, 1968), CuSOD (Steinman, 1985), Cu/Zn-SOD (Langford et al., 1996; Desideri and Falconi, 2003) and NiSOD (Wuerges et al., 2004).

In *S. pneumoniae*, two superoxide dismutases, namely SodA and SodB, have been identified. However, it has been observed that only SodA is involved in the response to oxidative stress (Yesilkaya et al., 2000). The expression of SodA was found to be upregulated in aerobic cultures, and a mutant lacking this enzyme exhibited reduced growth and increased susceptibility to paraquat, a compound that generates superoxide upon reacting with oxygen. These findings suggest that the primary function of SodA is to scavenge superoxide radicals (Figure 1). Furthermore, in a mouse model of intranasal infection, the *sodA* mutant displayed a slower multiplication rate in the lungs within the first 24 h and a delayed appearance in the bloodstream, indicating the contribution of SodA to the virulence of pneumococcal infections (Yesilkaya et al., 2000). The *sodA* gene exhibited a significant increase in expression levels, exceeding 10-fold, in the lung and brain tissues compared to the blood, suggesting its significance in the context of infection (Oggioni et al., 2006). Through the utilization of microarrays and virulence assays, genes that were expressed during mouse infection were identified, and it was proposed that SodA plays a crucial role in the colonization process of *S. pneumoniae* (Mahdi et al., 2015).

In addition to SodA, which serves as the primary defense against superoxide anions, *S. pneumoniae* also utilizes other enzymatic systems to combat ROS, such as the thioredoxin and glutathione systems. Within the thioredoxin family, the thiol peroxidase (or peroxiredoxin) TpxD plays a significant role in regulating H_2_O_2_ levels by facilitating its reduction (Figure 1). It has been observed that the expression of the *tpxD* gene is upregulated in the presence of exogenous H_2_O_2_. Consistently, the absence of the *tpxD* gene has been found to negatively impact the survival and growth of *S. pneumoniae* under these conditions (Hajaj et al., 2012, 2017). The regulatory network that controls the expression of the *tpxD* gene will be commented in the “Gene Regulation mechanisms” section.

In the presence of oxidative conditions, one of the most prevalent biochemical reactions that occurs within cells is the reversible oxidation of cysteine thiols. This particular reaction can serve as a signal transduction pathway for cells (Lo Conte and Carroll, 2013), making it the most significant posttranslational modification that arises from exposure to H_2_O_2_. The sulfenylation (or S-hydroxylation) of thiols by H_2_O_2_ is particularly relevant due to the oxidative burst induced by neutrophils. It has been demonstrated the essential role of TpxD and glutathione in protecting against proteome sulfenylation. As a consequence of exposure to H_2_O_2_, more than 50 proteins in the pneumococcus become sulfenylated (Lisher et al., 2017).

In general, the efficient removal of peroxides by peroxiredoxins is facilitated by appropriate reductants. For this purpose, the most commonly utilized system is the thioredoxin (Trx) system, which consists of a Trx, a Trx reductase, and NADPH as the electron source (de Oliveira et al., 2021; Figure 1). In *S. pneumoniae*, it has been demonstrated that the recombinant TpxD thioredoxin is functional using a Trx system derived from *Escherichia coli* (Hajaj et al., 2012). The pneumococcal genome encodes multiple thioredoxins and a Trx reductase similar to the one found in *Streptococcus mutans* (Marco et al., 2013). Although the pneumococcal TrxB enzyme has not been yet characterized, it is hypothesized that this enzyme likely plays a role in facilitating efficient catalysis by the thiol peroxidase TpxD.

The tripeptide glutathione, which is composed of γ-L-glutamyl-L-cysteinyl-glycine, is the most abundant non-protein thiol found in living organisms. It serves as a powerful intracellular antioxidant and is present in eukaryotes, proteobacteria, and a limited number of Gram-positive bacteria (Ku and Gan, 2019). In certain bacteria, the synthesis of glutathione is facilitated by enzymes such as γ-glutamylcysteine and glutathione synthetase. Glutathione is primarily utilized for various cellular processes, particularly in defense against ROS attacks. In this context, the reduced form of glutathione (GSH) is converted into a disulfide-bonded form (GSSG), and the balance between these two compounds is regulated by a GSH reductase named Gor, which employs NADPH as a reducing agent. The detoxification of ROS through glutathione can occur either through direct interaction with GSH or through the action of a GSH peroxidase that utilizes GSH to reduce H_2_O_2_ (Arenas et al., 2010; Figure 1).

*S. pneumoniae*, like other bacteria, lacks GSH biosynthetic enzymes. However, this pathogen does expresses GshT, an ABC transporter that allows the uptake of extracellular glutathione (Potter et al., 2012). The disruption of the *gshT* gene resulted in increased sensitivity to external H_2_O_2_, indicating its involvement in the oxidative stress response of *S. pneumoniae*. Furthermore, these genes have been found to play a role in pneumococcal pathogenesis, as mutants lacking these genes exhibited reduced virulence in a mouse model of infection (Potter et al., 2012).

An alternative strategy to counteract oxidative stress involves the direct reduction of oxygen levels within cells. Despite lacking heme proteins and cytochromes, *S. pneumoniae* utilizes NADH oxidase to detoxify oxygen. This enzyme catalyze its reduction of oxygen through NADH, resulting in the production of either H_2_O or H_2_O_2_, depending on the cellular redox state (Higuchi, 1992; Figure 1). A soluble form of NADH oxidase (Nox) has been identified and characterized in *S. pneumoniae.* Disruption of the *nox* gene, which encodes for NAD oxidase, has been found to abolish NADH oxidase activity, impair virulence and affect competence development in *S. pneumoniae*, while not compromising bacterial growth in aerobic conditions (Auzat et al., 1999; Echenique and Trombe, 2001). Indeed, other authors showed that the *nox* mutant, while displaying normal growth under limited aeration conditions, is incapable of growing exponentially in highly aerated conditions. This highlights the importance of NADH oxidase activity in enabling *S. pneumoniae* to cope with oxidative stress (Yu et al., 2001).

In eukaryotic organisms, there are transmembrane NADPH oxidases that share similar biochemical properties with NADH oxidases, but have a higher affinity for NADPH instead of NADH (Rotrosen et al., 1992). In the case of *S. pneumoniae*, it was reported for the first time as a bacterial NADPH oxidase. A recombinant form of this oxidase, also named Nox (or Nox2, to distinguish from the NADH oxidase), exhibited several biochemical characteristics that resembled those of human NOX2, which is considered the standard model for NOX enzymes. These similarities were observed in terms of oxygen consumption and the production of superoxide (Hajjar et al., 2017). NOX enzymes are commonly related to multicellularity functions that are characteristic of eukaryotic cells, but the physiological role of this NADPH oxidase in bacteria, particularly in the oxidative stress response, remains to be elucidated.

## Defense mechanism against antimicrobial oxidants

The heme peroxidases present in immune cells, particularly neutrophils, play a vital role in facilitating the reaction between H_2_O_2_ and pseudohalide (SCN^−^) or halide ions (Br-or Cl^−^) in various bodily fluids, including plasma, the oral cavity, and the digestive and respiratory tracts in humans. This reaction takes place during the oxidative burst of leukocytes and generates potent oxidizing agents such as hypochlorous acid (HOCl) and hypothiocyanous acid (HOSCN), which possess both bacteriostatic and bactericidal properties. These highly reactive compounds effectively eradicate bacteria, thereby playing a critical role in the antibacterial function of the innate immune system (Day, 2019; Arnhold and Malle, 2022).

**Figure 2:**
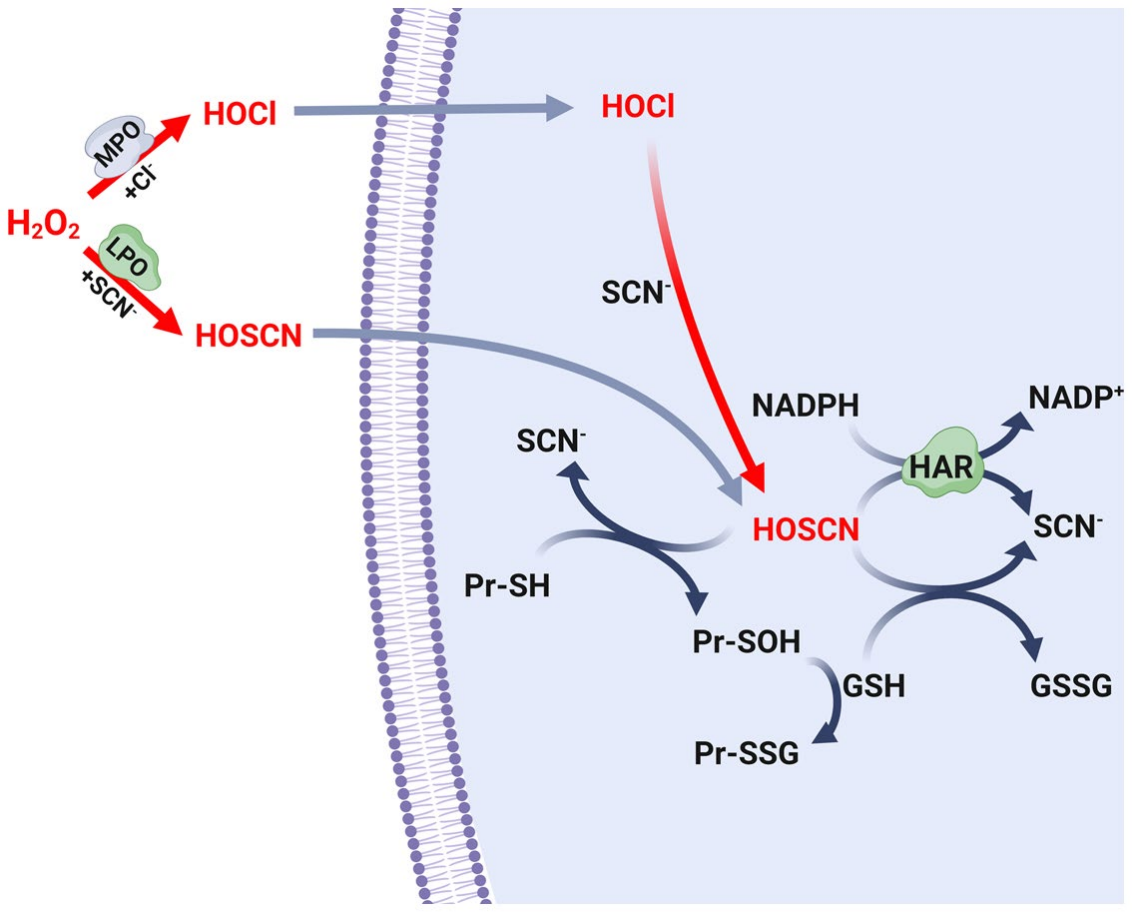
Biosynthesis and metabolism of antimicrobial oxidants. Scheme of the biosynthetic pathways of the hypochlorous acid (HOCl) and hypothiocyanous acid (HOSCN), as well as the defense mechanism of *S. pneumoniae* leaded by glutathione and the Har protein. SCN-, thiocyanate; MPO, myeloperoxidase; LPO, lactoperoxidase; GSH, reduced glutathione; GSSG, oxidized glutathione; Pr-SH, reduced protein thiols; Pr-SOH, sulfenic acid-modified proteins; Pr-SSG, glutathionylated proteins. GSSG formed in this scheme is reduced to GSH by glutathione reductase (Gor) using NADPH, as described in Figure 1.

Among the heme peroxidases responsible for the synthesis of antimicrobial oxidants, it is worth noting that myeloperoxidase (MPO) stands out as the sole enzyme capable of producing hypochlorous acid (HOCl) when released into autophagosomes during neutrophil phagocytosis (Paumann-Page et al., 2017). On the other hand, lactoperoxidase (LPO) is predominantly found in tears, breast milk, and saliva of mammals, and is recognized as the primary source of hypothiocyanous acid (HOSCN) (Ashby et al., 2009; Arnhold and Malle, 2022; Figure 2). Consequently, the LPO/H_2_O_2_/SCN-system, which generates HOSCN, is important for controlling the oral microbiome through the production of oxidants (Barrett and Hawkins, 2012; Courtois, 2021). In addition to the synthesis of antioxidants within cells, both myeloperoxidase (MPO) and lactoperoxidase (LPO) can be released from dying leukocytes into the extracellular space during inflammation (Winterbourn et al., 2016; Ramirez et al., 2018). In the presence of SCN^−^, these heme peroxidases may selectively produce HOSCN (Arnhold and Malle, 2022). Alternatively, eosinophil peroxidase (EPO) is directly released into extracellular environments due to its enzymatic function, which is involved in eliminating parasites that cannot be phagocytized. EPO reacts with halides, but it exhibits a preference for SCN-in order to synthesize HOSCN (Ramirez et al., 2018; Arnhold and Malle, 2022; Figure 2).

*S. pneumoniae* possesses the capacity to produce substantial quantities of H_2_O_2_ in order to eliminate other microorganisms found in the respiratory tract. However, the presence of SCN-plays a significant role in reducing the levels of H_2_O_2_ generated by both host and bacterial cells. Specifically, the enzymes LPO and MPO, which are present in the respiratory mucosa, convert SCN-to HOSCN (Figure 2). In an environment rich in chloride, *S. pneumoniae* has been observed to be susceptible to HOCl produced by MPO. Nevertheless, the pneumococcus develops resistance due to the presence of SCN^−^, which facilitates the conversion of HOCl to HOSCN (Figure 2; Shearer et al., 2022a). *In vitro* assays showed that the LPO/H_2_O_2_/SCN-system generated a lethal concentration of HOSCN for the pneumococcal strains TIGR4 (serotype 4) and EF3030 (serotype 19F) (Gingerich et al., 2020). In contrast, strains D39 (serotype 2) and SP264 (serotype 23) survived in cultures with a 20-fold higher concentration of HOSCN compared to those obtained with the LPO/H_2_O_2_/SCN-system. This discrepancy in survival can be attributed to the incubation time and the pneumococcal strains used. It is noteworthy that the pneumococcus exhibits inherent resistance to a concentration of HOSCN that is effective in eliminating other bacterial species, thereby giving it a clear advantage in evading host tissue defenses (Shearer et al., 2022a).

In *S. pneumoniae*, a strategy to reduce the levels of HOSCN involves the oxidation of GSH. HOSCN can directly oxidize the reduced thiol groups of proteins (Pr-SH) within the cell, forming sulfenic acids, while GSH is converted to GSSG. Accordingly, the sulfenic acid-modified proteins (Pr-SOH) can subsequently react with GSH to produce glutathionylated proteins (Pr-SSG) (Figure 2). In bacteria, it is important to note that both S-sulfenylation and S-glutathionylation are reversible post-translational modifications. However, the specific redoxins responsible for catalyzing these reverse reactions have yet to be identified and characterized in *S. pneumoniae*.

The role of the GSH transporter GshT and the GSH reductase Gor in the resistance mechanism of *S. pneumoniae* against HOSCN has also been investigated. Mutants lacking the *gshT* and *gor* genes have been found to exhibit increased sensitivity to exogenous HOSCN generated by the LPO/H_2_O_2_/SCN-system, indicating the crucial role of GSH in the HOSCN resistance mechanism of *S. pneumoniae* (Shearer et al., 2022c).

Furthermore, the identification and characterization of a flavoprotein disulfide reductase named Har (*h*ypothiocyanous *a*cid *r*eductase), which exhibits HOSCN reductase activity, has contributed significantly to the understanding of this mechanism. *In vitro* assays have shown that this enzyme utilizes NAD(P)H to reduce HOSCN to SCN-through a reaction similar to that described for thioredoxin reductases (Shearer et al., 2022b; Figure 2). The *har* mutant, when exposed to the LPO/H_2_O_2_/SCN-system, has shown that the HOSCN reductase is essential for pneumococcal viability in the presence of HOSCN. Nevertheless, the enzymatic activity of Har is complemented by the action of GSH as described earlier. The *har gshT* and *har gor* double mutants exhibited higher growth inhibition compared to the *har* single mutant (Shearer et al., 2022b).

## Manganese transport systems

Prokaryotic cells lack internal compartments and regulate metal cation flux across the cytoplasmic membrane to maintain metal homeostasis (Jakubovics and Jenkinson, 2001). Mn^2+^ is an essential cation for bacterial physiology and plays a central role in various cellular processes, including amino acid metabolism, glycolysis, nucleic acid degradation, protein cleavage, transformation, germination, and sporulation. Its primary function, however, lies in the oxidative stress response, where it assists in the detoxification of ROS as a critical cofactor for SodA (or MnSOD). This defense mechanism is likely to be widespread among bacteria and may also contribute to their virulence (Jakubovics and Jenkinson, 2001).

In *S. pneumoniae*, the uptake of Mn^2+^ is controlled by an ATP-binding cassette (ABC) cation permease consisting of three proteins: pneumococcal surface antigen A (PsaA), which acts as the Mn^2+^-specific solute binding component, and PsaB and PsaC. These proteins are encoded by the *psaBCAD* operon, with *psaD* (or *tpxD*) encoding for the TpxD thiol peroxidase. It is worth noting that the enzymatic activity of TpxD is not influenced by the concentration of Mn^2+^. The *psaD/tpxD* gene is transcribed separately from the other genes in the operon (Novak et al., 1998).

When the *psa* genes are inactivated, *S. pneumoniae* becomes completely dependent on externally added Mn^2+^ for normal growth (Dintilhac et al., 1997) and shows increased sensitivity to exogenous H_2_O_2_ (Tseng et al., 2002; McAllister et al., 2004). Additionally, the mutants lacking *psaA* and *psaD/tpxD* exhibited elevated expression of SodA and Nox, suggesting that PsaA and PsaD/TpxD may have important roles in regulating redox homeostasis in *S. pneumoniae* (Tseng et al., 2002). The regulation of the manganese transport genes will be discussed in the “Gene Regulation Mechanisms” section.

Furthermore, it has been documented that a deficiency in Mn^2+^ leads to a reduction in the transcription of *sodA*, suggesting that the expression of this gene is controlled through an unidentified pathway that responds to Mn^2+^ levels (Eijkelkamp et al., 2014). The *psaA* mutant showed a loss of virulence in otitis media, respiratory tract, sepsis and nasopharyngeal colonization in animal models, underscoring the importance of a functional Mn^2+^ transporter in the pathogenicity of the organism (Berry and Paton, 1996; Marra et al., 2002; McAllister et al., 2004).

In contrast to the Mn^+2^ uptake system, *S. pneumoniae* also has an Mn^+2^ exporter, named MntE, which is utilized by this pathogen to control possible high toxic levels of Mn^2+^ in the bacterial cytoplasm. The *mntE* mutant displayed heightened susceptibility to Mn^2+^ stress due to Mn^2+^ accumulation within the cell. Interestingly, this mutant strain also demonstrated increased resistance to oxidative stress, which was hypothesized to be a result of enhanced activity of Mn^2+^-dependent superoxide dismutase. The significance of the Mn^2+^ exporter in pneumococcal pathogenesis was determined through the observation of reduced virulence in the *mntE* mutant strain in a murine model of infection (Rosch et al., 2009). To prevent Mn^2+^ toxicity in the presence of elevated cytoplasmic Mn^2+^ levels, *S. pneumoniae* expresses another Mn^2+^ exporter known as MgtA, which belongs to the PII-type ATPase family of proteins. The expression of the *mgtA* gene is upregulated in response to Mn^2+^ stress, and this regulation is dependent on a Mn^2+^-sensing riboswitch belonging to the *yybP-ykoY* family (Martin et al., 2019).

Mn^2+^ is a vital trace element that is present in very low concentrations in the human body. In the context of bacterial pathogenesis, Mn^2+^ is found to be 1,000 times more concentrated in secretions compared to internal body sites. This significant difference in concentration may serve as a signal for bacterial cells to detect the transition from the mucosal surface to deeper tissues. It primarily circulates in the bloodstream bound to transferrin, with a smaller portion bound to albumin (Scheuhammer and Cherian, 1985).

Due to its importance in metabolism, the sequestration of Mn^2+^ has been found to impede bacterial growth. This process is a part of the host’s immune response to pathogens and is facilitated by the S100A8/S100A9 heterodimer calprotectin (CP), which has the ability to bind to Mn^2+^ and Zn^2+^ ions (Damo et al., 2013). In addition to its chemotactic and proinflammatory properties, CP exhibits antimicrobial characteristics. CP is prominently expressed by neutrophils, monocytes, and epithelial cells, and it is released into the sites of inflammation caused by bacterial infections (Striz and Trebichavsky, 2004). Notably, a significant release of CP has been observed during the course of pneumococcal infections (Raquil et al., 2008). In an infection model involving mice lacking S100A9, it was observed that pneumococcal pneumonia worsened (Ostermann et al., 2023). The sequestration of Mn^2+^ by CP plays a crucial role in the defense against infection and is a vital component of the innate immune response to bacterial pathogens.

Zn^2+^ is another essential element for bacterial metabolism, and pathogens rely on acquiring this metal from host tissues in order to thrive, establish colonization, and cause disease. Similar to the mechanism observed for Mn^+2^, the mammalian host employs a strategy of sequestering these cations to impede bacterial growth. This is achieved through the action of proteins belonging to the S100 family, such as CP and calgranulin, which are part of an innate immune response that withholds metals from invading bacteria (Hood and Skaar, 2012). On the contrary, mammalian hosts employ another antimicrobial tactic called metal intoxication, wherein high levels of Zn^2+^ are released into phagosomes to impair internalized bacteria (Sheldon and Skaar, 2019).

Regarding the metal homeostasis in *S. pneumoniae*, there exists an interplay between the metabolisms of Mn^2+^ and Zn^2+^. Elevated levels of Zn^2+^ have been found to inhibit growth, increase susceptibility to oxidative stress, and diminish virulence (Jacobsen et al., 2011; McDevitt et al., 2011; Eijkelkamp et al., 2014). The proposed mechanism involves the displacement of Mn^2+^ by Zn^2+^ in the PsaA protein, thereby hindering the uptake of Mn^2+^ and resulting in a deficiency of this cation within the cytoplasm (Jacobsen et al., 2011; Counago et al., 2014). Nevertheless, throughout the course of pneumococcal infection, both CP (Rosen et al., 2022) and calgranulin (Rosen and Nolan, 2022) have the ability to sequester Zn^2+^ from PsaA, facilitating the binding of Mn^2+^ to this protein and subsequent uptake of Mn^2+^. Interestingly, these proteins, which are employed by host cells as antimicrobial agents, paradoxically promote bacterial pathogenesis in this particular context.

## Iron metabolism

Iron is another key metal involved in bacterial oxidative stress. It is found in human tissues at low levels and is primarily transported in plasma by transferrin. Within immune cells, the reaction between Fe^2+^ and H_2_O_2_ leads to the formation of ROS through the Fenton reaction. These compounds cause DNA damage and exhibits high toxicity, ultimately resulting in the death of bacterial cells (Haschka et al., 2021).

In *S. pneumoniae*, it has been reported that three ABC transporters, namely PiuBCDA, PiaABCD, and PitADBC, play a role in regulating the intracellular iron levels (Brown et al., 2001a,b, 2002). On the other hand, the endogenous production of H_2_O_2_ facilitates the Fenton reaction (Pericone et al., 2003).

In the context of H_2_O_2_ production by the wild-type strain under aerobic conditions, a microarray analysis of *S. pneumoniae* revealed the upregulation of the *piuB/piuD* and *spxB* genes, results that were compared to the wild-type strain cultured under anaerobic conditions. This resulted in an elevation of Fe^2+^ and pyruvate oxidase levels in aerobic cultures, which subsequently stimulated the synthesis of endogenous H_2_O_2_. In consequence, the control of intracellular Fe^2+^ levels are essential for the OSR (Lisher et al., 2017). The regulation of the iron transport genes will be discussed in the “Gene Regulation Mechanisms” section.

Lipoproteins commonly serve as substrate-binding proteins for ABC transporters of many substrates, including sugars, cations, minerals, oligopeptides, amino acids, and polyamines, which are implicated in bacterial virulence. In *S. pneumoniae*, Lgt is indirectly involved in the uptake of metals, such as Fe^2+^, Mn^2+^, Zn^2+^ Ni^2+^, and Cu^2+^. The *lgt* mutant showed impaired ABC transport functions. For example, the use of paraquat revealed that this mutant is more susceptible to oxidative stress, and this phenotype is due to a decrease in cations such as Fe^2+^ and Mn^2+^. These cations contribute to the production of ROS and the OSR, respectively, as mentioned. This mutant also exhibited a significant impairment in nasopharyngeal colonization, sepsis, and pneumonia in mouse infection models, confirming the association between OSR and pathogenesis (Chimalapati et al., 2012).

In relation to iron metabolism, Dpr is a nonheme iron-containing ferritin that confers resistance to H_2_O_2_. It functions as a chelating protein that diminishes the free iron levels within the cell, thereby protecting pneumococcal cells from ROS generation by inhibiting the Fenton reaction. In a mouse model, the *dpr* mutant exhibited impaired virulence, specifically a deficiency in colonization (Hua et al., 2014).

Among the metal chelating systems cited to prevent intracellular ROS production, *S. pneumoniae* also possesses a flavin reductase, named FlaR, originally reported as PsipB (pneumococcal surface immunogenic protein B) (Ling et al., 2004). This enzyme not only has NADP reductase activity but also binds Fe^2+^ to protect the pneumococcus from oxidative stress through the reduction of free iron concentrations within the cytoplasm. Due to its surface localization, FlaR also has the capacity to act as a pneumococcal adhesin (Morozov et al., 2018).

## Repair systems

Bacteria not only require mechanisms to detoxify H_2_O_2_, but also repair systems to fix damaged molecules that are crucial for their survival under such stressful conditions. In the case of *S. pneumoniae*, a chaperone/protease known as HtrA (*h*igh-*t*emperature *r*equirement *A*) has been extensively studied. Initially identified as a heat shock protein, HtrA plays a vital role in the pathogenesis of *S. pneumoniae* (Gasc et al., 1998; Sebert et al., 2002). Through a signature-tagged mutagenesis screen, HtrA was identified as a virulence factor for *S. pneumoniae* (Hava and Camilli, 2002). This finding was further supported by studying an *htrA* mutant, which showed reduced virulence in a mouse model. However, in bacterial culture, this mutant exhibited slower growth at 42°C and increased sensitivity to oxidative stress, highlighting the importance of this chaperone in the OSR (Ibrahim et al., 2004).

In response to stressful conditions, certain bacteria employ a proteasome-like system that employs the ClpP protease to ensure their survival (Kahne and Darwin, 2021). This protease is coupled with hexameric ATPases, which facilitate ATP hydrolysis during the proteolysis process (Mahmoud and Chien, 2018). In *S. pneumoniae*, ClpP has been identified as a heat-shock protein that is crucial for survival during thermal and oxidative stress, as well as playing a significant role in virulence. Nevertheless, further studies are needed to understand the putative relationship between the ClpP function and OSR (Robertson et al., 2002). Analyzing a cellular infection model, it was observed that the *clpP* mutant exhibited heightened sensitivity to oxidative stress in macrophages (Park et al., 2010). In Gram-positive bacteria, ClpL is an ATPase that works in conjunction with ClpP to degrade proteins (Choi et al., 1999). The pneumococcal ClpL is a unique member of the Hsp100 family that is dependent on Mn^2+^ and exhibits chaperone activity without requiring co-chaperones (Park et al., 2015). Similar to ClpP, ClpL has also been classified as a heat-shock protein (Choi et al., 1999; Kwon et al., 2003).

In our lab we found that the *clpL* and *psaB* genes, which encodes for the ClpL chaperone and the subunit B of the Mn^2+^ transporter, PsaB, respectively, are regulated by SirR (Reinoso-Vizcaino et al., 2020), and this will be commented in the “Gene Regulation Mechanisms” section. The *clpL* mutant showed greater susceptibility to external exposure to H_2_O_2_, which is a similar finding to that observed in the *psaB* mutant (McCluskey et al., 2004). It is known that the presence of Mn^2+^ enhances the hydrolase and chaperone activities of ClpL, and this feature has not been reported for other proteins in the HSP100 family (Park et al., 2015). We propose that the deficiency in Mn^2+^ uptake observed in the *psaB* mutants is responsible for altering the enzymatic activity of SodA and ClpL. These enzymes are dependent on intracellular Mn^2+^ levels and are essential for the pneumococcal OSR (Reinoso-Vizcaino et al., 2020).

One potential consequence of exposure to ROS is the oxidation of methionine residues to methionine sulfoxide, which must be repaired in order for bacterial survival to occur (Saleh et al., 2013). Thioredoxins, in addition to their role in scavenging H_2_O_2_, are widely distributed oxidoreductases that contribute to repair systems by providing reducing equivalents to methionine sulfoxide reductases (Msr). These enzymes reduce oxidized methionines (Ezraty et al., 2005) or directly repair oxidized methionines (Collet and Messens, 2010). In *S. pneumoniae*, the TrxA and MsrAB1 thioredoxins have been identified, but a linking with OSR has not yet been determined (Kim et al., 2009). TlpA, also known as Etrx1, is an enzyme belonging to the thiol-specific antioxidant (TlpA/TSA) family (Marchler-Bauer et al., 2017) that has thioredoxin properties. The *tlpA* mutant exhibited increased susceptibility to external H_2_O_2_, which correlates with the observed thioredoxin activity displayed by the recombinant TlpA protein. In terms of pneumococcal pathogenesis, the *tlpA* mutation had more detrimental effects on later stages of infection compared to the initial stages in a mouse model (Andisi et al., 2012). The operon containing the *tlpA* (or *etxR1*) gene exhibited increased transcriptional activity in response to oxidative stress. This operon also facilitates the expression of genes encoding the cytochrome C-type biogenesis protein, CcdA1, and the methionine sulfoxide reductase, MsrAB2. These proteins, collectively referred to as CTM (an abbreviation for CcdA, TlpA, and MsrAB), play a vital role in the cellular response to oxidative stress. The MsrAB2 enzyme is particularly important in protecting against oxidative stress by converting methionine sulfoxide residues back to methionine. This conversion process is facilitated by the presence of thioredoxins. In a different region of the pneumococcal genome, a group of genes was identified that includes paralogues of CcdA1 and Etrx1, named CcdA2 and Etrx2. The authors of the study demonstrated that both Etrx1 and Etrx2 thioredoxins, which are exposed on the cell surface, along with their corresponding redox partners, CcdA1, CcdA2, and MsrAB2, are important for defending against exposure to ROS outside the cell and for the development of *S. pneumoniae* infections (Gennaris and Collet, 2013; Saleh et al., 2013; Ribes et al., 2016).

## Polyamine metabolism

Among all the diverse factors that impact the OSR, bacterial metabolism is probably one of the most unexplored aspects. Polyamines, for example, are essential for regulating many cellular processes in bacteria. During its biosynthetic process, the enzyme lysine decarboxylase CadA contributes to the conversion of lysine to cadaverine (Nakamya et al., 2018). In *S. pneumoniae*, a metabolomics analysis of the *cadA* mutant revealed that altered polyamine levels indirectly led to reduced expression of the trehalose phosphotransferase system (Ayoola et al., 2019). Trehalose is a disaccharide that has been shown to scavenge free radicals and provide protection against oxidative stress in yeast (Jiang et al., 2019) as well as in pathogenic mycobacteria (Kalscheuer and Koliwer-Brandl, 2014). Based on these findings, it is hypothesized that CadA-mediated trehalose metabolism is crucial for the oxidative stress response (Ayoola et al., 2019) and for virulence in *S. pneumoniae* (Shah et al., 2011).

PotABCD is a transporter responsible for the uptake of putrescine and spermidine implicated in polyamine metabolism. The *potABCD* mutant exhibits heightened vulnerability to oxidative stress, akin to the *cadA* mutant, due to a notable decline in polyamine and trehalose levels, which serve as antioxidants. Metabolomics analysis further elucidated that the *potABCD* mutant displays diminished glutathione levels, providing additional evidence to support its observed phenotype (Nakamya et al., 2021).

In the pathway of polyamine synthesis, the enzyme SpeA, which is an arginine decarboxylase, contributes in the production of agmatine (Ayoola et al., 2020). Similar to the observed effects of deletions in *cadA* and *potABCD*, the null mutant of *speA* has been found to increase susceptibility to oxidative stress. Transcriptomic and proteomic analysis determined that this mutation negatively affects the expression of various genes known to be essential for OSR, including *tpxD* and *htrA*. Additionally, the authors propose that SpeA not only regulates the synthesis of glutathione but also influences the endogenous production of H_2_O_2_ (Nakamya et al., 2021). The *speA* mutation also leads to an increase in carbon flow through the pentose phosphate pathway (PPP), which is considered a cellular response to oxidative stress. This metabolic route helps maintain the balance of NADH/NADPH redox and generates ribose-5-phosphate, thereby enhancing nucleotide synthesis and preventing DNA damage caused by ROS (Stincone et al., 2015). These findings highlight the significance of maintaining polyamine homeostasis in the physiology of bacterial pathogens. Furthermore, it is central to understand the relationship between specific metabolic pathways and the OSR in *S. pneumoniae*.

## Gene regulation mechanisms

**Figure 3:**
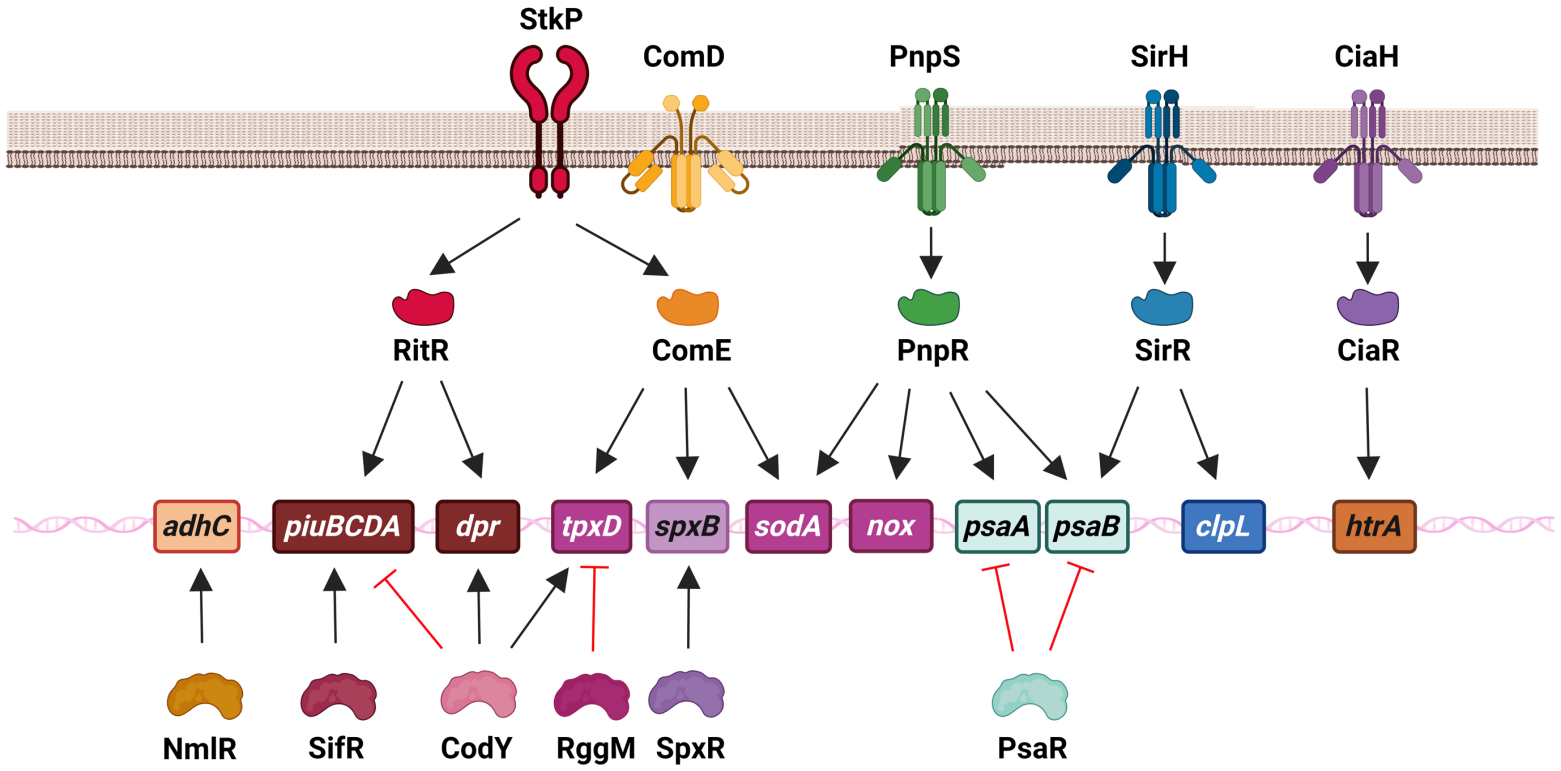
Regulatory systems that control genes involved in the oxidative stress response of *S. pneumoniae* in both extracellular and intracellular survival. This network is constituted by the serine–threonine protein kinase StkP, three two-component systems, including SirRH (TCS01), PnpSR (TCS04), and CiaRH (TCS05), as well as individual regulators, such as ComE (without its cognate kinase ComD) and RitR that belong to the family of response regulators, and NmlR, SifR, CodY, RggM, and SpxR. In the scheme, these regulatory systems control different genes that encode enzymes that contribute to iron transport (*piuBCDA* and *dpr*), manganese transport (*psaAB*), ROS detoxification (*sodA* and *tpxD*), H_2_O_2_ production (*spxB*), alcohol dehydrogenase (*adhC*) and repair mechanisms (*clpL* and *htrA*).

The pneumococcal OSR is mediated by a diverse array of proteins, suggesting that their expression is controlled by various regulatory systems to ensure the survival of this pathogen (Figure 3). Bacterial gene expression is tightly regulated by proteins that detect changes in the environment and initiate a physiological response. The pneumococcal genome displayed the presence of numerous individual regulators, as well as two-component systems (TCS), which are a common mechanism for signal transduction in bacteria (Gomez-Mejia et al., 2018). In *S. pneumoniae*, there have been 13 reported TCS, each consisting of a response regulator and its corresponding histidine kinase. The only exception is RitR, which is an orphan response regulator (Paterson et al., 2006; Gomez-Mejia et al., 2018). Notably, seven of these regulatory systems are involved in regulating oxidative stress adaptation, in addition to individual regulators. This highlights the importance of gene regulation in this adaptive mechanism of *S. pneumoniae* (Figure 3).

In the presence of a high level of Fe^2+^, the transcription of the *piuBCDA* genes is repressed by RitR (*r*epressor of *i*ron *t*ransport) (Ulijasz et al., 2004; Ong et al., 2013). This particular orphan regulator undergoes phosphorylation by the serine–threonine kinase StkP (Figure 3). Additionally, it interacts with PhpP, which acts as a dephosphorylating agent for StkP and this interaction indirectly affects the activation state of RitR (Echenique et al., 2004; Ulijasz et al., 2009). It has been demonstrated that RitR phosphorylation promotes the expression of the *piu* genes (Ulijasz et al., 2009). Notably, a *ritR* mutant exhibited increased susceptibility to H_2_O_2_, which was found to be correlated with a higher intracellular concentration of free iron. This observation was attributed to the derepression of the *piu* operon, which may be responsible for the heightened toxicity resulting from ROS hyperproduction through the Fenton reaction (Ulijasz et al., 2004).

RitR has been found to have an additional regulatory role in sensing high levels of H_2_O_2_ through a single cysteine. When ROS levels are low, RitR in its reduced monomeric form weakly binds to the *piuBCDA* promoter, thereby facilitating the expression of this operon. However, under high ROS levels, the Cys^128^ residue of RitR undergoes oxidation, leading to the dimerization of RitR and its subsequent binding to the *piuBCDA* promoter. This results in the repression of transcription of the *piu* operon, thereby preventing iron transport and protecting pneumococci from the toxic effects of the Fenton reaction (Glanville et al., 2018). Contrary to the repressor effect showed on the expression of *piuBCDA*, RitR activates the transcription of *dpr*. In both cases, this regulator is essential to decrease the levels of free iron (Ulijasz et al., 2004; Figure 3).

In the realm of individual regulators, a transcriptional repressor belonging to the Rrf2 family has been identified and studied in *S. pneumoniae*. Even in the presence of endogenous H_2_O_2_, this repressor, known as SifR (streptococcal IscT-like family transcriptional repressor), is capable of detecting quinones. This ability enables *S. pneumoniae* to effectively utilize Fe^2+^/catecholamine complexes derived from the host, which are acquired through the iron transporter PiuBCDA. Under low Fe^2+^ levels, SifR interacts with free catechol-derived quinones and disengages from the DNA promoter region, resulting in the activation of the SifR regulon. This regulon includes *piuBCDA*, among other genes, thereby facilitating an increase in iron uptake. These findings demonstrate that SifR is part of a complex regulatory system that controls the OSR in *S. pneumoniae* (Zhang et al., 2022; Figure 3).

CodY is a regulatory protein that controls many cellular processes and metabolic pathways. Its ability to bind to DNA is influenced by changes in the levels of branched-chain amino acids (Brinsmade et al., 2010). It was also reported that CodY, a regulator previously associated with nutritional gene regulation in *S. pneumoniae* (Kloosterman et al., 2006; Hendriksen et al., 2008), is capable of activating the expression of *tpxD*, which encodes the TpxD thiol peroxidase. In response to H_2_O_2_, CodY is activated through the oxidation of two cysteines that trigger a conformational change in this regulator, enhancing DNA binding and activating the expression of *tpxD* (Hajaj et al., 2017). In addition, CodY activates the expression of the *dpr* gene, which encodes a protein involved in iron storage and peroxide resistance, while repressing the *fat (or piu)* genes, which encode proteins responsible for iron transport (Hendriksen et al., 2008; Figure 3).

The *codY* gene has been found to be essential for the survival of *S. pneumoniae*. The *codY* mutant was only obtained by suppressing mutations that inactivated the genes encoding FatC (or PiuC) and AmiC. These genes are involved in iron and oligopeptide transport, respectively, and their expression is repressed by CodY (Caymaris et al., 2010; Johnston et al., 2015). It has been proposed that the unviability of the *codY* mutant may be attributed to iron toxicity. This is because the *codY* mutation leads to the derepression of *fat (or piu)* genes and the inhibition of *dpr* expression, resulting in increased iron levels and subsequent oxidative stress through the Fenton reaction (Hendriksen et al., 2008; Caymaris et al., 2010; Figure 3).

In *S. pneumoniae*, the NmlR regulator was reported to activate the expression of the *adhC* gene, which encodes the alcohol dehydrogenase AdhC, when bacteria were exposed to formaldehyde and methylglyoxal. The *nmlR* mutant displayed resistance to high levels of oxygen in cultures, a phenotype that was attributed to lower production of H_2_O_2_, although the mechanism remained unclear (Potter et al., 2010; Figure 3).

In relation to the detrimental effects of antimicrobial oxidants, the amino acids cysteine and methionine are most commonly oxidized by HOCl in proteins, resulting in protein unfolding and bacterial death (Ulfig and Leichert, 2021). A comprehensive analysis of the bacterial cells treated with sublethal concentrations of HOCl was conducted using RNA-seq transcriptome analysis. This analysis demonstrated that the oxidant significantly stimulated the expression of various regulons associated with diverse functions, including OSR (NmlR, SifR, CTM operon), proteostasis (CtsR, HrcA), and metal stress response (SczA, CopY), among others. Conversely, other regulons linked to OSR, such as CiaR, RitR, and CodY were found to be downregulated (Fritsch et al., 2023), and their implications are discussed in this section. It is worth noting that the CTM operon encompasses the genes responsible for encoding CcdA, EtrxA1, and MsrAB2, while the CtsR operon governs the expression of the genes encoding ClpL and ClpP. The functions of all these proteins have been discussed in the “Repair Systems” section. These findings underscore the intricate nature of the response to HOCl and offer valuable insights into the cellular damage inflicted by this potent oxidant.

In light of the significant score achieved in the RNA-seq transcriptome analysis, the NlmR transcriptional regulator was selected for further investigation as a potential candidate for detecting the presence of HOCl. Subsequently, it has been experimentally proven that NmlR possesses the ability to sense HOCl by means of the oxidation of Cys^52^. This oxidative reaction activates the transcription of the *nmlR-adhC* operon *in vivo*. It is evident that NmlR plays an important role in the OSR, enabling *S. pneumoniae* to survive during macrophage infections. Apparently, AdhC is a metabolic enzyme whose expression is controlled by NmlR under oxidative conditions. This enzyme provides resistance to HOCl and ROS stress, however, the specific role of AdhC in ROS detoxification has not yet been elucidated (Fritsch et al., 2023; Figure 3).

As previously stated, the SpxB pyruvate oxidase is a significant virulence factor that primarily synthesizes endogenous H_2_O_2_ and plays a crucial role in the OSR of the pneumococcus (Pericone et al., 2003). Through a screening of morphological mutants, it was discovered that point mutations in a specific gene led to reduced H_2_O_2_ production in *S. pneumoniae*, resulting in alterations in the pneumococcal capsule. This gene, subsequently named *spxR* (pyruvate oxidase regulator), encodes a regulator capable of activating the expression of *spxB* and, consequently, the endogenous production of H_2_O_2_ in response to the metabolic state (Ramos-Montanez et al., 2008; Figure 3).

RggM (or Rgg1952) is a transcriptional regulator that belongs to the Rgg family and controls the regulation of *tpxD* gene expression in *S. pneumoniae* (Bortoni et al., 2009). In the presence of oxygen, a mutant lacking the *rggM* gene did not exhibit susceptibility to H_2_O_2_, and the expression of *tpxD* was higher in the *rggM* mutant compared to wild-type cells. TpxD is an important enzyme involved in detoxification, and the overexpression of *tpxD* in the *rggM* mutant is responsible for its phenotype under oxidative conditions. This suggests that RggM negatively regulates the expression of *tpxD* (Bortoni et al., 2009; Figure 3).

In our laboratory, we have characterized a new signaling pathway consisting of the serine–threonine kinase StkP (Echenique et al., 2004) and the response regulator ComE, which belongs to ComDE (TCS12) (Claverys et al., 2006). This transcription factor is typically activated through phosphorylation at an Asp^59^ residue by its cognate histidine kinase ComD. ComD is the natural receptor of CSP (competence-specific peptide) and is activated when CSP accumulates in the extracellular space through a quorum sensing mechanism (Claverys et al., 2006). Under acidic conditions, ComE is activated through phosphorylation of the Thr^128^ residue by a crosstalk event with StkP. This activation allows ComE to control the expression of over 100 genes, indicating that the StkP/ComE pathway induces global changes in response to stress. Through transcriptomic and qPCR assays, it was revealed that the StkP/ComE pathway activates the expression of the *spxB*, *tpxD*, and *sodA* genes. The non-phosphorylatable *comE^T128A^* mutant, as well as the *comE* and *stkP* mutants, exhibited an impaired oxidative stress response. It was proposed that this phenotype was due to reduced expression of the *tpxD*, *sodA*, and *spxB* genes (Pinas et al., 2018). Previous studies had reported that a mutant lacking the *stkP* gene displayed decreased *tpxD* expression, but the underlying mechanism was not elucidated (Echenique et al., 2004; Nováková et al., 2005; Sasková et al., 2007). In this sense, we propose that StkP/ComE represents an alternative pathway for activating *tpxD* expression (Pinas et al., 2018; Figure 3).

Regarding the regulation of OSR mediated by TCSs, the expression of the *htrA* gene that encodes for the chaperone HtrA is regulated by CiaRH (Sebert et al., 2002; Mascher et al., 2003; Halfmann et al., 2007). Indeed, the deletion of *ciaR* mutant lead to a reduced HtrA expression and, consequently, to an increased susceptibility to external H_2_O_2_ similar to that of the *htrA* mutant. However, when HtrA was overexpressed, this phenotype was restored (Ibrahim et al., 2004; Figure 3).

Through a systematic screening of mutants defective in TCS, and a subsequent comparative RNAseq analysis, we found that TCS01, also known as SirRH (stress-induced response), regulates the expression of *clpL* and *psaB*, as discussed in the “Repair Systems” section. These genes are responsible for encoding the ClpL chaperone and the PsaB Mn^2+^ transporter, respectively. The *sirR*, *clpL*, and *psaB* mutants exhibited the same phenotypes as the *comE* and *stkP* strains. This is due to the absence of ClpL chaperone function or a decrease in Mn^2+^ levels, which negatively affects both SodA and ClpL since their enzymatic activities rely on Mn^+2^ levels (Reinoso-Vizcaino et al., 2020; Figure 3).

In relation to the regulation of Mn^2+^ homeostasis, TCS04 has been identified as a key regulator that activate the transcription of the *psaBCA* operon. This was evidenced by microarray assays comparing the *rr04* mutant, which lack the response regulator of this TCS, with the wild-type strain. TCS04 showed to be related to OSR due to the diminished oxidative stress response observed in the *rr04* mutant (McCluskey et al., 2004). The same phenotype was displayed by mutants in the *tcs09* genes, which encode TCS09. The probable mechanism by which TCS09 is involved in resistance against oxidative stress is mediating the regulation of carbohydrate metabolism, cell wall integrity and amount of capsular polysaccharide (Hirschmann et al., 2021; Figure 3).

In *S. pneumoniae*, the metal-dependent regulator PsaR has been observed to inhibit the transcription of the *psaBCA* operon in the presence of excess Mn^2+^ ions (Lisher et al., 2013). Microarray assays conducted using a *psaR* mutant strain demonstrated the upregulation of the *psaBCA* operon (Hendriksen et al., 2009). Notably, the *psaR* mutant exhibited reduced virulence in pulmonary infections in a mouse model, suggesting that Mn^2+^ ions serve as a signal for the expression of virulence factors in different host environments (Johnston et al., 2006). This study highlights the involvement of several transcriptional regulators in the gene regulation of OSR. PsaR belongs to a group of regulators that indirectly influence OSR by regulating the uptake of Mn^2+^ ions through the *psaBCA* genes, which are also regulated by TCS04 (McCluskey et al., 2004). This regulatory network underscores the significance of maintaining Mn^2+^ homeostasis in the OSR of *S. pneumoniae* (Figure 3).

## Intracellular survival mechanism

It has been reported that *S. pneumoniae* can be internalized into host cells through various mechanisms, including clathrin- (Radin et al., 2005) and caveolin-dependent endocytosis (Gradstedt et al., 2013; Asmat et al., 2014) and dynamin-independent endocytosis (Agarwal et al., 2010). The presence of *S. pneumoniae* within vesicles that are labeled with classical endocytic markers, such as early, late, and recycling markers, as well as lysosomal markers, suggests that the bacteria are degraded through the endocytic pathway (Radin et al., 2005; Agarwal et al., 2010). On the other hand, it has been provided evidence that the autophagic pathway can also be effective in killing *S. pneumoniae* (Li et al., 2015; Ogawa et al., 2018).

For decades, *S. pneumoniae* has been considered as a prototypical extracellular pathogen. Nevertheless, recent research has revealed that this particular bacterium has the ability to survive within the host cells, despite the mechanisms that host cells employ to protect themselves against it. For instance, pneumococci have been observed within the vacuoles of intact A549 pneumocytes, as well as freely in the cytoplasm of damaged cells (Talbot et al., 1996). Similarly, when pneumococci invade brain microvascular endothelial cells, the vesicles containing the bacteria are recycled to the apical surface and can also migrate to the basolateral cell surface, enabling viable pneumococci to cross epithelial and endothelial cells (Ring et al., 1998). In these same type of cells, *S. pneumoniae* has been found to survive for extended periods of time. This survival is enhanced by the inhibition of lysosomal activity, suggesting that the majority of intracellular pneumococci are killed within vesicles that fuse with lysosomes (Gradstedt et al., 2013). When pneumococci are internalized by macrophages and dendritic cells, they are enclosed in vacuoles that are coated with MRC-1, a mannose receptor. These vacuoles do not merge with lysosomal compartments, allowing *S. pneumoniae* to survive within the host cells. This intracellular survival enables the bacteria to use macrophages and dendritic cells as Trojan horses to spread the infection (Subramanian et al., 2019). A similar phenotype was reported by other authors, who described that *S. pneumoniae* survive entrapped in autophagosomes (Ogawa et al., 2018), while this pathogen showed to be able to replicate in murine splenic macrophages (Ercoli et al., 2018). In HL-1 cardiomyocytes, *S. pneumoniae* was also found in vacuoles and it was confirmed that this pathogen replicates intracellularly, indicating that this survival mechanism is essential for the early events during cardiomyocyte invasion. Furthermore, it was shown that pneumolysin and SpxB-derived H_2_O_2_ production were necessary for cardiomyocyte killing (Brissac et al., 2018).

To ensure their survival within host cells, particularly immune cells, bacteria must possess the ability to withstand challenging conditions, including oxidative stress. The pneumococcal capsule is widely recognized as a crucial virulence factor that aids pneumococci in evading phagocytosis by immune cells (Feldman and Anderson, 2020). However, once internalized, this capsule assumes an antioxidant role, safeguarding the bacteria against intracellular destruction caused by the host’s production of ROS. Additionally, the capsule has been found to enhance the efficiency of translocation across vascular endothelial cells (Brissac et al., 2021).

In our laboratory, we conducted experiments that revealed the ability of *S. pneumoniae* to survive for extended periods within cell lines such as human A549 pneumocytes and murine RAW 264.7 macrophages. This survival mechanism was dependent on the CiaR and ComE response regulators (Pinas et al., 2008; Cortes et al., 2015). Further, we have also investigated the StkP/ComE signaling transduction system and its role in response to acidic stress and oxidative genes (Figure 3). We found that this system controls the expression of *tpxD*, *sodA*, *spxB*, and *murN*, which are involved in various cellular processes such as cell wall biosynthesis and autolysis. Activation of the StkP/ComE pathway leads to the upregulation of these genes, and a disbalance on MurN provokes alterations in the cell membrane biosynthesis and consequent autolysis. We observed that mutants lacking *comE* and *stkP* showed increased survival within A549 pneumocytes, possibly due to the inhibition of autolysis and H_2_O_2_ production. This is likely caused by low expression levels of *murN* and *spxB*, respectively. Furthermore, we propose that the StkP/ComE pathway is crucial for *S. pneumoniae* to survive acidic and oxidative stress within host cells (Pinas et al., 2018). Interestingly, it was also discovered that SpxB-mediated H_2_O_2_ production enhances the intracellular survival of *S. pneumoniae* by inactivating lysosomal cysteine cathepsins, which affects the degradative capacity of lysosomal vesicles in brain microvascular endothelial cells. This process facilitates the movement of pneumococci across cellular compartments (Anil et al., 2021).

## Synergism between influenza A and *Streptococcus pneumoniae*

Another factor that contributes to the development of pneumococcal infections is the combined effect that occurs when patients are infected with both influenza A and *S. pneumoniae*. It is well-known that individuals previously infected with influenza often suffer additional bacterial infections that complicate their treatment, and *S. pneumoniae* is one of the most commonly found bacterial pathogens in these cases (Sender et al., 2021). To gain a better understanding of this synergistic mechanism, we conducted a study to examine how influenza A infection affects the ability of *S. pneumoniae* to survive inside lung cells. We focused on the M2 protein of influenza A virus (IAV), which has been shown to inhibit the fusion of autophagosomes with lysosomes (Gannage et al., 2009). Our hypothesis was that this characteristic promotes the survival of *S. pneumoniae* within autophagosomes (Reinoso-Vizcaino et al., 2020).

Influenza A infection is associated with an elevation in ROS levels within infected cells. This increment is produced by an upregulation of the transcripts that encodes for the NADH oxidase 4 (NOX4), with a consequent overexpression of this enzyme (Amatore et al., 2015), and by the interaction of the viral M2 protein with MAVS (mitochondrial antiviral signaling protein) on mitochondria, causing overproduction of ROS (Wang et al., 2019). The overproduction of ROS can lead to lung tissue damage and the activation of inflammatory cells. The imbalance of the redox environment facilitates the transmission of the influenza virus between cells by making host epithelial cells more susceptible (Liu et al., 2017). To survive under these conditions in pneumocytes, *S. pneumoniae* must induce an oxidative stress response, and we have confirmed that influenza infection increases ROS production in pneumocytes. In this viral-bacterial synergy, we have discovered that *S. pneumoniae* senses, either directly or indirectly, cellular changes induced by IAV through a TCS named SirRH. This system regulates the expression of more than 170 genes. In particular, we analyzed the *clpL* and *psaB* genes, which encode a protein chaperone and an Mn^+2^ transporter, respectively, as mentioned above. The *sirR*, *clpL*, and *psaB* showed increased susceptibility to oxidative stress and did not exhibit any improvement in intracellular survival when pneumocyte cells were infected with IAV. These results suggest that the SirRH two-component system is capable of sensing stress conditions induced by IAV and controlling adaptive responses that enable the survival of *S. pneumoniae* in IAV-infected pneumocytes (Reinoso-Vizcaino et al., 2020).

In a mouse coinfection model, it has been observed that the growth of *S. pneumoniae* under oxidative stress conditions induced by influenza is dependent on the expression of the protease/chaperone HtrA (Sender et al., 2020). This requirement is mediated by the presence of CiaR, a response regulator that governs the expression of the *htrA* gene (Sebert et al., 2002). At present, only SirRH and CiaR have been described as direct players implicated in this synergistic mechanism. However, considering the intricate nature of regulating the OSR, it is probable that other signal transduction systems are involved in this regulatory network.

## Antibiotic persistence

The endogenous synthesis of H_2_O_2_, facilitated by the enzyme SpxB, is also implicated in the mechanism of action of fluoroquinolones (FQs), a class of antibiotics commonly used to treat pneumococcal infections. In addition to their ability to inhibit DNA gyrase, FQs also induce metabolic changes that lead to an increase in pyruvate levels. Subsequently, SpxB converts this pyruvate, resulting in a concentration of H_2_O_2_ that is more than 20 times higher. Concurrently, FQs also stimulate the expression of genes involved in iron transport, which are encoded in the *piuBCDA* operon, leading to elevated levels of cytoplasmic iron. The combination of increased iron and H_2_O_2_ levels conducts to a significant rise in hydroxyl radical production through the Fenton reaction (Figure 3). This can cause damage to proteins, lipids, and DNA. Consequently, it has been postulated that ROS contribute to the bactericidal effect of FQs on *S. pneumoniae* (Ferrandiz et al., 2016).

During pneumococcal infections, immune cells generate inflammatory foci that produce oxidative environments. In our study, we have investigated the potential influence of these stressful conditions on the mechanism of action of fluoroquinolone antibiotics (FQs). It is well-established that bacteria can develop mechanisms to tolerate lethal concentrations of bactericidal antibiotics, under stressful conditions. When this phenomenon occurs in a bacterial subpopulation that shows a different killing rate that the rest of cells, the persisters cannot replicate in the presence of antibiotics, this capacity is not inherited, and it is induced by stress conditions, this mechanism is named “triggered persistence” (Michiels et al., 2016; Balaban et al., 2019). Different stress conditions have been identified as capable of triggering persistence, with starvation being the most commonly observed among them (Nguyen et al., 2011). Additionally, oxidative stress has been found to be another environmental factor that can induce antibiotic persistence in bacterial (Hong et al., 2012; Vega et al., 2012; Wu et al., 2012).

**Figure 4:**
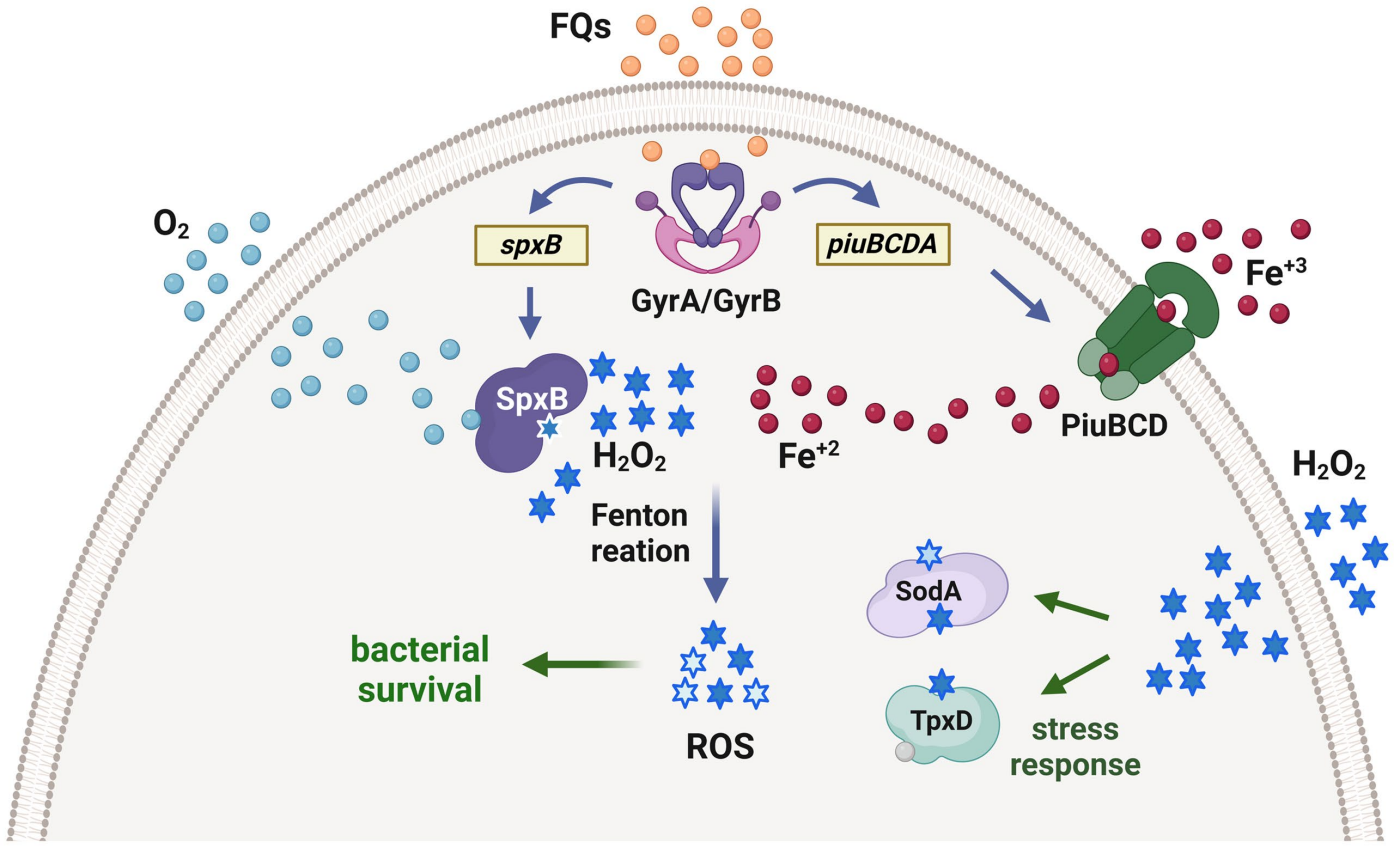
Proposed mechanism of fluoroquinolone persistence induced by oxidative stress in *S. pneumoniae*. Fluoroquinolones inhibit the topoisomerases in the bacteria, leading to the upregulation of the *piuBCDA* genes. These genes are responsible for enhancing iron uptake, which subsequently increases the levels of intracellular Fe^+2^. Additionally, the SpxB pyruvate oxidase synthesizes endogenous H_2_O_2_. The interaction between Fe^+2^ and H_2_O_2_ through the Fenton reaction generates ROS, which cause oxidative damage to DNA, proteins, and lipids. This cellular impairment contributes to bacterial death caused by fluoroquinolones. Interestingly, prior exposure of pneumococci to H_2_O_2_ within host cells triggers the expression of enzymes that detoxify ROS, such as the thiol peroxidase TpxD and the superoxide dismutase SodA. These enzymes are part of an adaptive response that protects pneumococci from oxidative damage induced by fluoroquinolones and facilitates the generation of fluoroquinolone persisters, which are capable of surviving in the presence of these antibiotics [adapted from Ferrandiz et al. (2016)].

Our findings revealed that oxidative stress induces a state of persistence in *S. pneumoniae* when exposed to FQs. This phenomenon was observed not only in bacterial cultures but also during the temporary intracellular existence of pneumococci within pneumocytes, macrophages, or neutrophils. To our knowledge, this is the first reported mechanism of antibiotic persistence in *S. pneumoniae* (Hernandez-Morfa et al., 2022). Exposure to oxidative stress was found to upregulate the expression of two enzymes, SodA and TpxD, which provide protection against ROS induced by FQs and enhance the intracellular survival of *S. pneumoniae* (Figure 4). Importantly, other authors have validated our research outcomes using clinical strains of *S. pneumoniae*. They found persistence to multiple antibiotics, including fluoroquinolones, although the underlying mechanisms were not elucidated (Geerts et al., 2022).

Based on our findings, we propose that persistence may have implications for the effectiveness of antibiotic treatment and could be a component of a multi-step process in the development of fluoroquinolone resistance (Hernandez-Morfa et al., 2022).

## Conclusion

Throughout the process of evolution, both aerobic and facultative aerobic/anaerobic bacteria have developed mechanisms to tolerate ROS. This adaptation is crucial for protecting themselves from oxidative stress-induced cellular damage. Bacterial pathogens, specifically those capable of surviving within host cells, have also developed protective systems to counteract the ROS released by immune cells in the extracellular space of infected tissues.

*S. pneumoniae* is known for its exceptional ability to produce H_2_O_2_, and this capacity allows pneumococci to be part of the human nasopharynx microbiota. This bacterium utilizes this metabolic weapon to eliminate other microorganisms that cannot survive in such oxidative conditions (Pericone et al., 2000). However, if the production of H_2_O_2_ is not properly controlled, it can lead to a significant imbalance of oxidative stress, ultimately causing detrimental effects on the physiology of *S. pneumoniae*. In consequence, *S. pneumoniae* has evolved various protective systems that enable its survival under oxidative stress conditions. Overcoming this initial hurdle of oxidative stress is essential for *S. pneumoniae* to successfully establish an infection.

We have underscored the intricate nature of the stress response mechanisms employed by *S. pneumoniae* for self-defense. The presence of multiple systems suggests that these mechanisms may be utilized independently or in conjunction with one another. However, it remains unclear whether this adaptability serves as a redundant safeguard to ensure an appropriate response, functions as a sequential step in a cascade of reactions, or represents a specific reaction to a particular oxidative environment. The OSR in *S. pneumoniae* is a multifaceted process that involves various regulatory mechanisms, such as two-component systems and individual regulators. These mechanisms are capable of sensing changes in redox state and initiating a physiological response to mitigate the detrimental effects of ROS on cellular components.

The majority of these investigations were conducted either *in vitro* or in animal models. Additionally, we have carried out studies that elucidate the OSR during the intracellular life cycle of *S. pneumoniae*, as well as the significance of this mechanism in the observed synergistic effect during coinfection. It is our belief that the development of novel methodological approaches is imperative in order to comprehend how this pathogen regulates the intracellular attack of ROS, particularly within immune cells.

From a therapeutic standpoint, one of the most crucial discoveries pertaining to oxidative stress is its capacity to induce persistence to fluoroquinolones in host cells. In this context, the transient intracellular existence of *S. pneumoniae* may potentially create a favorable environment for the emergence of fluoroquinolone resistance, as has been suggested for other bacterial pathogens (Klugman, 2003; Dalhoff, 2012).

Finally, this revision not only underlines the complexity of the oxidative stress response and its relevance for the pneumococcal pathogenesis, but also unveils specific areas that require further investigation in the future. For example, there is a need to explore the regulatory mechanism of the oxidative stress response when the bacteria are present within immune cells. Moreover, it is crucial to examine the potential consequences of H_2_O_2_-induced FQ persistence on the effectiveness of antimicrobial treatment for pneumococcal infectious diseases. Further investigation is necessary to ascertain whether this persistence mechanism is an essential step in the evolution of antibiotic resistance.

## Author contributions

MH-M: Conceptualization, Writing – review & editing. NO: Conceptualization, Writing – review & editing. VZ: Conceptualization, Writing – review & editing. GP: Conceptualization, Writing – review & editing. NR-V: Conceptualization, Writing – review & editing. MC: Conceptualization, Writing – review & editing. MN-F: Conceptualization, Writing – review & editing. PC: Conceptualization, Writing – review & editing. JE: Conceptualization, Writing – review & editing, Funding acquisition, Writing – original draft.

## Funding

The author(s) declare financial support was received for the research, authorship, and/or publication of this article. This work was supported by the National Agency of Scientific and Technological Promotion (ANPCYT; IP-COVID-19 240; FONCYT-PICT-2016-#0805; FONCYT-PICT 2018-#2046-Prestamo BID, to JE), and the National University of Cordoba (SECYT-UNC-2020, to JE). JE is a member of the Research Career of the CONICET. MH-M and NO are recipients of CONICET PhD fellowships, and VZ is the recipient of a ANPCYT PhD fellowship.

## Conflict of interest

The authors declare that the research was conducted in the absence of any commercial or financial relationships that could be construed as a potential conflict of interest.

## Publisher’s note

All claims expressed in this article are solely those of the authors and do not necessarily represent those of their affiliated organizations, or those of the publisher, the editors and the reviewers. Any product that may be evaluated in this article, or claim that may be made by its manufacturer, is not guaranteed or endorsed by the publisher.

## Footnotes

^1^ https://www.cdc.gov/pneumococcal/global.html
